# Expression of EMT-Related Genes CAMK2N1 and WNT5A is increased in Locally Invasive and Metastatic Prostate Cancer

**DOI:** 10.7150/jca.34564

**Published:** 2019-10-15

**Authors:** Isa Carneiro, Filipa Quintela-Vieira, João Lobo, Catarina Moreira-Barbosa, Francisco Duarte Menezes, Ana Teresa Martins, Jorge Oliveira, Regina Silva, Carmen Jerónimo, Rui Henrique

**Affiliations:** 1 Cancer Biology and Epigenetics Group, Research Center of Portuguese Oncology Institute of Porto (GEBC CI-IPOP), R. Dr. António Bernardino de Almeida, 4200-072, Porto, Portugal;; 2 Department of Pathology, Portuguese Oncology Institute of Porto (IPO Porto), R. Dr. António Bernardino de Almeida, 4200-072, Porto, Portugal;; 3 School of Health, Polytechnic of Porto (ESS), R. Dr. António Bernardino de Almeida 400, 4200-072 Porto, Portugal;; 4 Department of Urology, Portuguese Oncology Institute of Porto (IPO Porto), R. Dr. António Bernardino de Almeida, 4200-072, Porto, Portugal;; 5 Department of Pathology and Molecular Immunology, Institute of Biomedical Sciences Abel Salazar, University of Porto (ICBAS-UP), Rua Jorge Viterbo Ferreira 228, 4050-513, Porto, Portugal

**Keywords:** Prostate cancer, epithelial-to-mesenchymal transition, prognosis, *CAMK2N1*, * WNT5A*

## Abstract

**Purpose:** Prostate cancer (PCa) varies clinically from very indolent, not requiring therapeutic intervention, to highly aggressive, entailing radical treatment. Currently, stratification of PCa aggressiveness is mostly based on Gleason score, serum PSA and TNM stage, but outcome prediction in an individual basis is suboptimal. Thus, perfecting pre-therapeutic discrimination between indolent and aggressive PCa, avoiding overtreatment is a major challenge. Epithelial to mesenchymal transition (EMT) allows epithelial cells to acquire mesenchymal properties, constituting a critical step in tumor invasion and metastization. Thus, we hypothesized that EMT-related markers might allow for improved assessment of PCa aggressiveness. **Methods and Results:** Using RealTime ready Custom Panel 384 assay, 93 EMT-related genes were assessed in normal prostate tissues (NPT, n=5), stage pT2a+b-PCa (n=5) and stage pT3b-PCa (n=5), from which *CAMK2N1*, *CD44*, *KRT14*, *TGFβ3* and *WNT5A* genes emerged as the most significantly altered. Expression levels were then evaluated in a larger series (16 NPT and 94 PCa) of frozen tissues using quantitative RT-PCR. Globally, *CAMK2N1*, *CD44* and *WNT5A* displayed higher expression levels at higher stages and less differentiated PCa. CAMK2N1 and WNT5A immunoexpression analysis disclosed significantly lower expression in NPT and increasing proportion of high-expression cases from pT2a+b to pT3b and metastatic PCa. Furthermore, higher *CAMK2N1* and *WNT5A* transcript levels associated with shorter disease-free and disease-specific survival. In multivariable analysis, a trend for *WNT5A* expression levels to independently predict DFS was disclosed (p=0.056). **Conclusions:** Globally, our findings suggest an association between PCa aggressiveness and increased expression of *CAMK2N1* and *WNT5A*, reflecting the acquisition of effective EMT characteristics by PCa cells.

## Introduction

Prostate cancer (PCa) is the second most frequently diagnosed cancer and the fifth leading cause of cancer-related death among men worldwide, being the most frequently diagnosed male cancer in developed countries 1. Currently, PCa management and treatment is decided based on serum prostate-specific antigen (PSA) levels, stage, histopathological characteristics and patient's life expectancy, among other factors 2. However, PCa behavior is frequently unpredictable, ranging from very indolent, amenable to active surveillance, to highly aggressive, requiring radical treatment 3. Whereas organ-confined PCa is mostly curable, locally or systemically advanced disease is uncurable as no effective curative treatments are available 2. Due to the inability to accurately distinguish tumors that will be harmless from those that will be lethal in many cases, physicians and patients tend to elect treatments with curative intent, entailing undesirable side-effects without a clear survival benefit 4, 5, 6, 5, 7. Thus, improved characterization of tumor aggressiveness is critical for reducing both overtreatment and mortality and it should be ideally based on the biological properties of PCa cells.

Epithelial-to-mesenchymal transition (EMT) is a process that has been associated with tumor aggressiveness, playing a central role in tumor invasion and metastasis 8, 9. EMT is a biologic process that allows epithelial cells to undergo multiple biochemical changes enabling the acquisition of a mesenchymal phenotype, thus enhancing migratory capacity, invasiveness, resistance to apoptosis, and augmenting the production of extracellular matrix components 10. During this transition, epithelial cells progressively loose expression of typical epithelial markers, such as E-cadherin and keratins, and gain expression of typical mesenchymal markers, including vimentin, α-smooth muscle actin (SMA) and N-cadherin 8, 10. EMT endows cancer cells with mobility and the capacity to invade tissues and organs, as well as entering blood vessels 8. After intravasation, these cancer cells are transported through the circulation and can leave the blood stream at a remote site where they may form metastasis 8. Considering the important role of EMT in tumor progression and metastasis formation, EMT-related markers might be used to weigh tumor aggressiveness, allowing for improved PCa patient management. Hence, we sought to identify EMT-related genes involved in PCa progression, through evaluation of their expression levels in a series of PCa patients submitted to radical prostatectomy, and further assess their clinical usefulness for prediction of disease progression.

## Material and Methods

### Clinical Samples

PCa tissue samples were prospectively collected from patients with clinically localized disease, consecutively diagnosed and treated with radical prostatectomy at Portuguese Oncology Institute of Porto, Portugal (2001-2012). All patients were treated and followed-up by the same multidisciplinary team of this Institute. To allow for group comparisons, cases enrolled comprised either pT2a+b (representing early stage disease) or pT3b (representing locally advanced disease) PCa, encompassing a total of 94 tumor samples. Cases with insufficient clinical information were discarded from the analysis. Samples of morphologically normal prostate tissue (NPT, n=16) were collected from the peripheral zone of prostates obtained in the context of cystoprostatectomy performed in males with bladder cancer. Only specimens that did not harbor PCa nor prostatic intraepithelial neoplasia (PIN), confirmed after thorough sectioning and histological observation (and use of immunohistochemistry ancillary studies, whenever necessary), were included in the study and served as controls. Also, patients previously subjected to chemotherapy due to bladder cancer in the neoadjuvant setting were excluded.

All tissue samples were promptly frozen immediately after surgery, following patient informed consent, and stored at -80ºC for further analysis. After histological confirmation of PCa and NPT by a dedicated uropathologist in 5µm-thick stained section, fresh-frozen tissue fragments were trimmed to maximize the yield (>70%) of target cells. Subsequently, an average of fifty 12µm thick sections were cut and every fifth sections a 5 µm section was stained to ensure a uniform percentage of target cells and to exclude contamination from neoplastic cells in normal tissue samples.

Secondly, formalin-fixed paraffin-embedded (FFPE) tissue samples from the same surgical specimens, collected for routine histopathological evaluation, were also used in this study. Regarding the macroscopic handling, prostate glands were totally embedded for histological examination and fragments were mapped according to the specific protocol of the Pathology Department. Given the greater availability of these FFPE samples compared to fresh frozen tissues, the series was further enriched in NPT samples (to the original 16 samples with fresh frozen tissue available already included, 29 FFPE samples were added, derived from further cystoprostatectomy specimens fulfilling the same requirements). Consultation cases were excluded. Gleason score 11, as well as pathological stage 12 of all PCa cases were assessed in histological slides from FFPE tissues fragments routinely collected from the same surgical specimens.

Thirdly, a set of metastatic PCa tissues (MET, n=57; 27 bone metastases, 18 lymph node metastases and 12 metastases from other regions) were also selected for immunoexpression analysis. Cases without sufficient amount of tumor cells and/or artifacts (namely in bone biopsies) were excluded. Relevant clinical data was retrieved from clinical charts.

This study, as well as the use of samples and the access to clinical data, was approved by the institutional review board (Comissão de Ética para a Saúde) of Portuguese Oncology Institute of Porto, Portugal (CES-IPOFG_EPE 019/08).

### RNA Extraction

Total RNA was extracted from fresh frozen samples using TRIzol method. Briefly, samples were suspended in TRIzol® reagent (Invitrogen) and, after homogenization using rotor-shaker, chloroform (Merck) was added. RNA was purified from the upper/aqueous phase of TRIzol extract using the PureLinkTM RNA Mini Kit (Invitrogen) according to the manufacturer's instructions. RNA concentration and purity ratios were then evaluated using NanoDrop ND-1000 spectrophotometer (ThermoScientific, Wilmington, DE, USA). Additionally, RNA integrity was checked by electrophoresis in 2% agarose-gel.

### Screening of 93 EMT Related Genes Expression

Based on a comprehensive literature review, 93 potential EMT-related genes were selected for gene expression evaluation (Table S1). Five NPT and ten PCa samples representing the tumor spectrum in this series considering pathological stage [pT2a+b-PCa (n=5) and pT3b-PCa (n=5)] were chosen for this analysis.

cDNA was synthesized from 1000ng of total RNA by reverse transcription using Transcriptor High Fidelity cDNA Synthesis Kit v.6 (Roche Applied Science, Mannhein, Germany), according to manufacturer's instructions.

Expression levels of 93 EMT-related genes were evaluated using RealTime ready Custom Panel 384 assay (Roche Applied Science, Mannhein, Germany). The QRT-PCR reaction was performed in a RealTime termocycler LightCycler® 480 II (Roche Applied Science, Mannhein, Germany), following manufacturer's instructions. Each experiment was performed in triplicates for PCa samples and without replicates for NPT.

The amount of mRNA of the genes studied was normalized to that of *TFRC* reference gene and the median value of NPT, pT2a+b-PCa and pT3b-PCa samples was chosen to calculate fold-difference in gene expression among groups, using the comparative Ct method (2^-ΔΔCt^). The 2^-∆∆Ct^ values inferior to 0.5 were considered to indicate a significant expression reduction, whereas 2^-∆∆Ct^ values above 2.0 indicated significant increase in expression. From the 93 EMT related genes analyzed, five genes were selected as potential markers of EMT in PCa based on differential expression between pT3b-PCa, NPT and pT2a+b-PCa, and correlation with gene behavior described in literature.

### Validation of Selected Genes

To validate the selected genes, expression levels were evaluated in a larger series of NPT (n=16) and PCa frozen tissue samples [pT2a+b-PCa (n=48) and pT3b-PCa (n=46)].

cDNA was synthesized from 300ng of total RNA using TransPlex® Whole Transcriptome Amplification (WTA) Kit (Sigma-Aldrich®) according to manufacturer's protocol. WTA reaction products were purified using QIAquick PCR Purification Kit (QIAGEN), according to manufacturer's protocol.

mRNA levels were evaluated using TaqMan® Gene Expression assays (Applied Biosystems) for the selected genes and for the endogenous control GUSβ. The QRT-PCR assay was performed in 96-well plates on an Applied Biosystems 7500 Real-time PCR system (Applied Biosystems), according to the recommended protocol. All samples were run in triplicate and two water blanks were added to each plate as negative controls. Serial dilutions of WTA-cDNA synthesized from prostate total RNA were analyzed as standards, allowing the construction of a standard curve for relative quantification and PCR efficiency assessment, for each plate. To determine the relative expression levels for each sample, mean quantity of each gene was normalized with mean quantity of endogenous control GUSβ.

### Immunohistochemistry

CAMK2N1 and WNT5A expression was assessed by immunohistochemistry in 3µm sections of FFPE samples [45 NPT, 94 PCa (pT2a+b and pT3b) and 57 MET]. The technique was performed using anti-CAMK2N1 goat polyclonal antibody (Santa Cruz Biotechnology Inc.; sc-161427) at 1:75 dilution with ImmunoCruz goat ABC Staining System (Santa Cruz Biotechnology Inc.; sc-2023), and anti-WNT5A mouse monoclonal antibody (abcam; 3D10-ab86720) at 1:2500 dilution with Nonolink^TM^ Max Polimer Detection System (Leica Biosystems). Antigen retrieval was performed by microwaving the sections at 800W for 20-30 minutes in Citrate buffer, for both markers. Normal brain was used as positive control for CAMK2N1 and lung adenocarcinoma for WNT5A. Immunoexpression was scored as 1 (weak expression ≤ 50% of cells), 2 (weak expression ≥ 50% of cells or moderate expression ≤ 50% of cells) or 3 (moderate expression in > 50% of cells or intense expression, typically > 50% of cells).

A flow-chart of the sample selection, cohorts used and including and excluding criteria is presented in Supplementary Figure 1.

### In silico validation in an independent cohort

To validate transcript and protein results obtained in our own tissue cohort we performed an *in silico* analysis of the publicly available The Cancer Genome Atlas (TCGA) database, by using the online resource cBioPortal for Cancer Genomics 13. mRNA data regarding both CAMK2N1 and WNT5A was imported from the online database, after which statistical analysis was performed.

### Statistical analysis

Samples were divided into categories according to pTNM (NPT, pT2a+b-PCa and pT3b-PCa, MET) and GS (GS≤6; GS 3+4=7; GS 4+3=7; GS=8; GS=9-10).

The Shapiro-Wilk's W-test allowed for the examination of the appropriateness of a normal distribution assumption for each of the parameters (data not shown).

Differences in expression levels of the EMT-related genes between the different groups of samples were firstly analyzed using Kruskal-Wallis one-way ANOVA, followed by non-parametric Mann-Whitney U-test, when appropriate. A non-parametric Spearman test was performed to assess correlation between gene expression levels and serum PSA and age.

The prognostic significance of available clinical variables (pathological stage, GS, age and serum PSA levels) was assessed by constructing disease-specific (DSS) and disease-free survival (DFS) curves using Kaplan-Meier method with log-rank test (univariable analysis). A Cox-regression model comprising the four variables (multivariable analysis) was also constructed. DFS was calculated from the date of the radical prostatectomy to the date of biochemical relapse, or date of last follow up, or death if relapse-free. For the purposes of survival analyses, all cases were coded based on the expression levels of each gene using percentile 75 value as empirical threshold. Cases were also subdivided according to serum PSA levels (below and above median values) and age (above 60, between 60 and 70, and above 70 years). Immunoexpression within groups of samples was compared using the Chi-square test, and to measure the strength and direction of associations, Somers' D test was additionally performed.

For the *in silico* analysis samples were grouped as close as possible to the categories already analyzed in the tissue cohort: pT2 vs pT3b tumors; GS=6 vs GS=7 vs GS=8 vs GS≥9; and additionally the comparison between pN0 and pN1 cases. Distribution of continuous variables between groups was also compared using the nonparametric Mann-Whitney U-test. Survival curves were also plotted using the Kaplan-Meier method and log rank test was used for survival analysis.

The level of significance was set at p<0.05. For multiple comparisons, Bonferroni's correction and Dunn's test were used to adjust p-values. Statistical analysis was performed using IBM® SPSS® Statistics 23 software and GraphPad Prism 6.

## Results

### Clinical Samples

For the purposes of this study, NPT and PCa tissue samples were used to evaluate transcript levels and protein expression of EMT-related genes. Relevant clinical and pathological data are summarized in Table 1. No significant differences in age distribution between NPT and PCa groups were found.

### Expression of EMT-related genes and clinicopathological correlates

Expression levels of 93 EMT-related genes were evaluated in five NPT and ten PCa samples. According to the previously described criteria, *CAMK2N1*, *CD44*, *KRT14*, *TGFβ3* and *WNT5A* were selected for validation. Array results disclosed decreased expression of *CD44*, *KRT14* and *TGFB3* in PCa-pT3b compared to PCa-pT2a+b, whereas the opposite was depicted for *WNT5A* and *CAMK2N1* (Table 2).

Expression levels of the selected five EMT-related genes were then assessed in a larger series of 15 NPT and 94 PCa frozen tissue samples. Comparing PCa to NPT, statistically significant differences were confirmed for *CD44* (p=0.013), *KRT14* (p=0.004) and *WNT5A* (p<0.001), but not for *CAMK2N1* and *TGFB3*.

A significant correlation between *CAMK2N1* expression levels, but not *CD44*, *KRT14*, *TGFB3* and *WNT5A*, and serum PSA levels of PCa patients was found (r=0.221, p=0.034).

**Figure 1:**
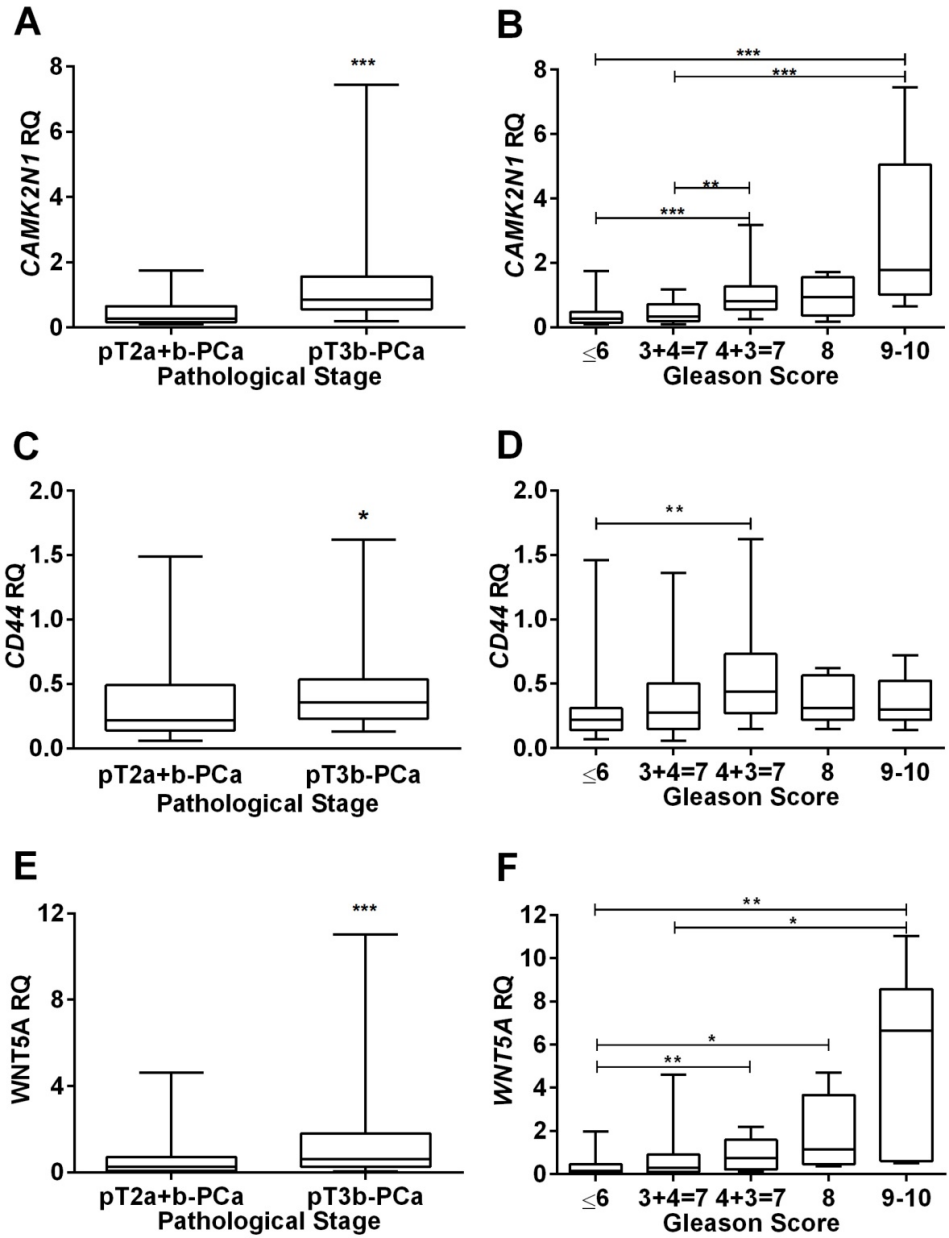
Association of *CAMK2N1*, *CD44* and *WNT5A* relative expression levels with clinicopathological data. Distribution of *CAMK2N1*(**A**), *CD44*(**C**) and *WNT5A*(**E**) expression levels according to pathological stage, showing higher expression levels in more advanced tumors, pT3b-PCa. Distribution of *CAMK2N1*(**B**), *CD44*(**D**) and *WNT5A*(**F**) expression levels according to Gleason Score, showing higher expression levels associated with a higher Gleason Score, mainly for *CAMK2N1* and *WNT5A*.

Concerning pathological stage, *CAMK2N1* (p<0.001), *CD44* (p=0.020) and *WNT5A* (p<0.001) expression levels were significantly higher in locally advanced (pT3b-PCa) compared to organ-confined (pT2a+b-PCa) disease (Figure 1A, C and E), but no significant differences for *KRT14* and *TGFB3* were depicted.

Analysis of variance indicated significant differences in expression levels among the five GS categories for *CAMK2N1* (p<0.001), *CD44* (p=0.015) and *WNT5A* (p<0.001). Globally, highest expression levels were found in higher GS categories and pair-wise comparisons, using Mann-Whitney U-test, *CAMK2N1* and *WNT5A* revealed significant differences among GS categories (Figure 1B, D and F).

### Survival analyses

The median follows up period of this series of primary PCa patients was 91 months (range: 3-161 months). At the time of the last follow-up, six patients (6.4%) had died from PCa and 24 (25.5%) developed biochemical recurrence. In 17 patients, serum PSA levels >0.2ng/mL persisted following surgery and these were not further considered for disease-free survival (DFS) analysis. As expected, pT3b PCa patients experienced significantly reduced DFS compared to pT2a+b PCa (p=0.032), and GS=8 / GS=9-10 PCa also associated with shorter time to relapse compared to the remainder categories (p<0.001). The same trend was observed for DSS [higher pathological stage (p=0.02) and GS category (p<0.001) associated with decreased DSS].

**Figure 2:**
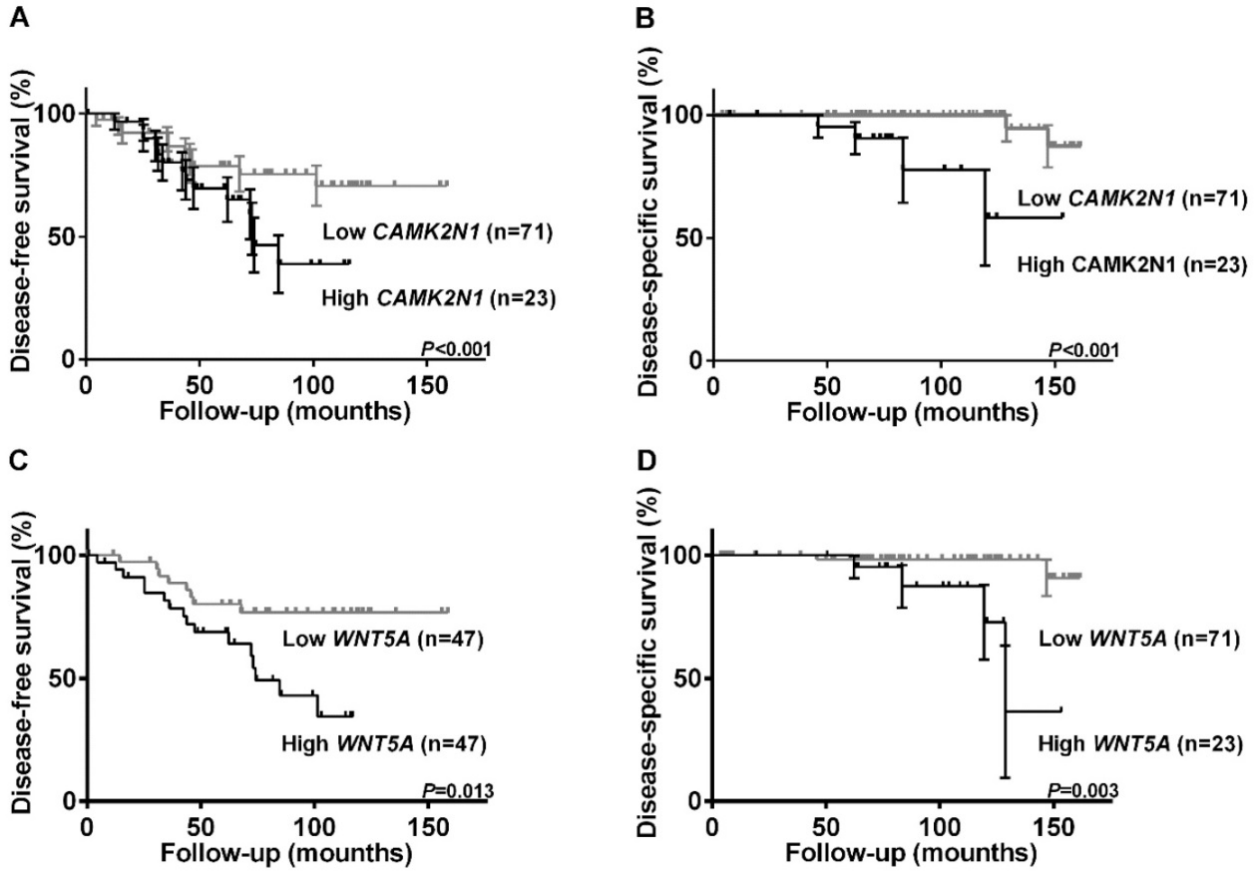
Kaplan-Meier estimated disease-free survival and disease-specific survival curves of 94 PCa patients. Disease-free survival curves according to relative expression levels of *CAMK2N1*(**A**) and *WNT5A* (**C**); Expression levels were categorized using third quartile (75^th^ percentile) value as the cutoff. Disease-specific survival curves according to relative expression levels of *CAMK2N1*(**B**) and *WNT5A* (**D**); Expression levels were categorized using second quartile (50^th^ percentile) value as the cutoff.

Higher *CAMK2N1* (Figure 2A and 2C) and *WNT5A* (Figure 2B and 2D) expression levels associated with shorter DFS (p=0.045 and p=0.013, respectively) and DSS (p<0.001 and p=0.003, respectively). No significant associations were found for *CD44*.

In multivariable analysis, *CAMK2N1* and *WNT5A* did not disclose independent prognostic value, although a trend for *WNT5A* expression levels independently predict DFS was disclosed (p=0.056).

### CAMK2N1 and WNT5A immunoexpression in primary and metastatic PCa

**Figure 3:**
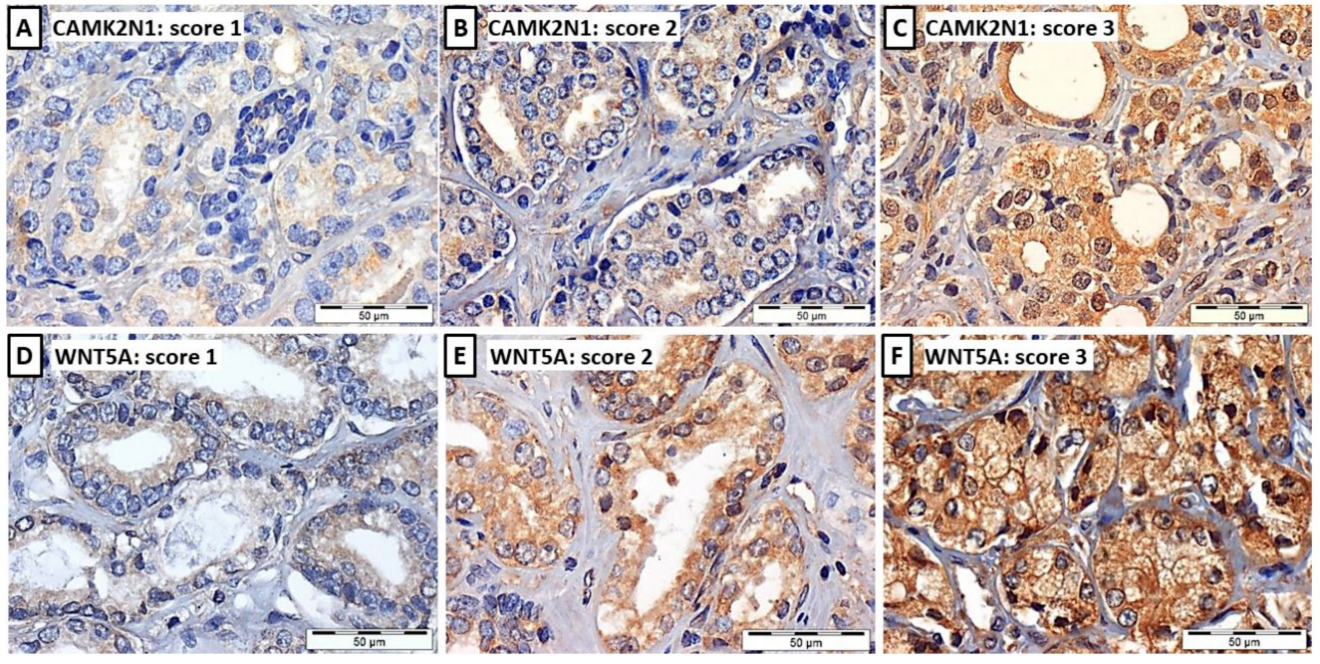
Illustrative images of CAMK2N1 (**A**, **B** and **C**) and WNT5A (**D**, **E** and **F**) immunoexpression in PCa tissues, considering the 3-tiered immunoscore: immunoscore 1 (**A** and **D)**, weak expression ≤ 50% of cells, immunoscore 2 (**B** and **E**), weak expression ≥ 50% of cells or moderate expression ≤ 50% of cells, and immunoscore 3 (**C** and **F**), moderate expression in > 50% of cells or intense expression, typically > 50% of cells.

**Figure 4:**
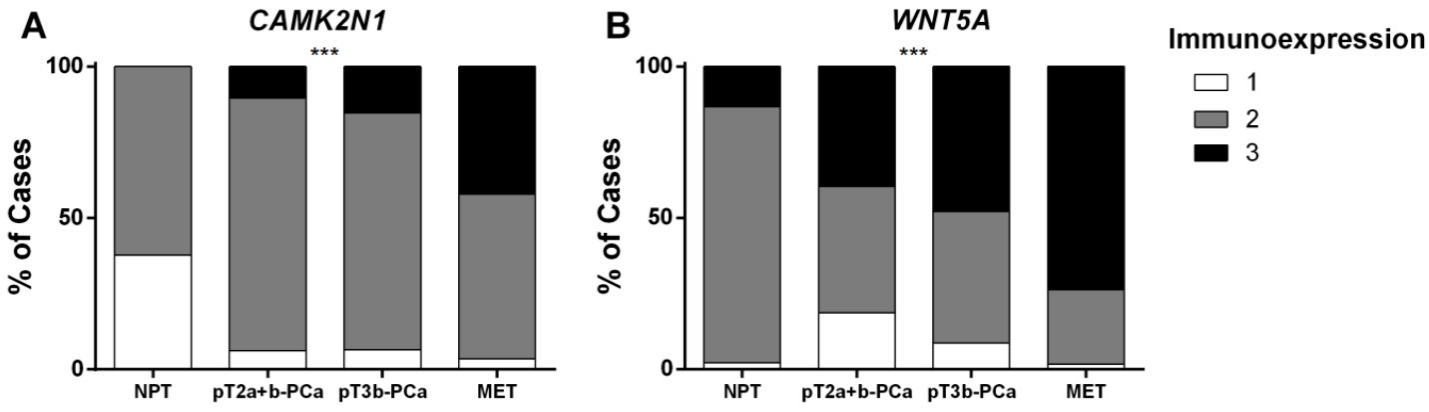
Immunoexpression of CAMK2N1 and WNT5A in prostate tissue samples (Cohort#3). Distribution among Cohort#3 groups of (**A**) CAMK2N1 immunoexpression and (**B**) WNT5A immunoexpression.

CAMK2N1 and WNT5A immunoexpression assessment was carried out in 45 NPT, 94 PCa (pT2a+b and pT3b; Figure 3) and 57 PCa metastases (MET). These two genes were selected owing to the correlation found with pathological features and survival. Somers' D test disclosed significant differences in expression for both genes (Figure 4A and 4B) (p<0.001, for both). Lowest expression was displayed by NPT whereas an increase in the proportion of cases with higher expression was apparent from PCa-pT2a+b to PCa-pT3b and MET, for both CAMK2N1 and WNT5A (CAMK2N1: v=0.418, p<0.001; WNT5A: v=0.347, p<0.001).

A similar trend was apparent for WNT5A, but not CAMK2N1, immunoexpression according to GS category, with higher categories depicting higher proportion of cases with high immunoexpression (Somers' D: v=0.227, p=0.013).

Survival analyses, however, did not disclose significant associations between CAMK2N1 or WNT5A immunoexpression and prognosis.

### In silico validation in the TCGA database

**Figure 5:**
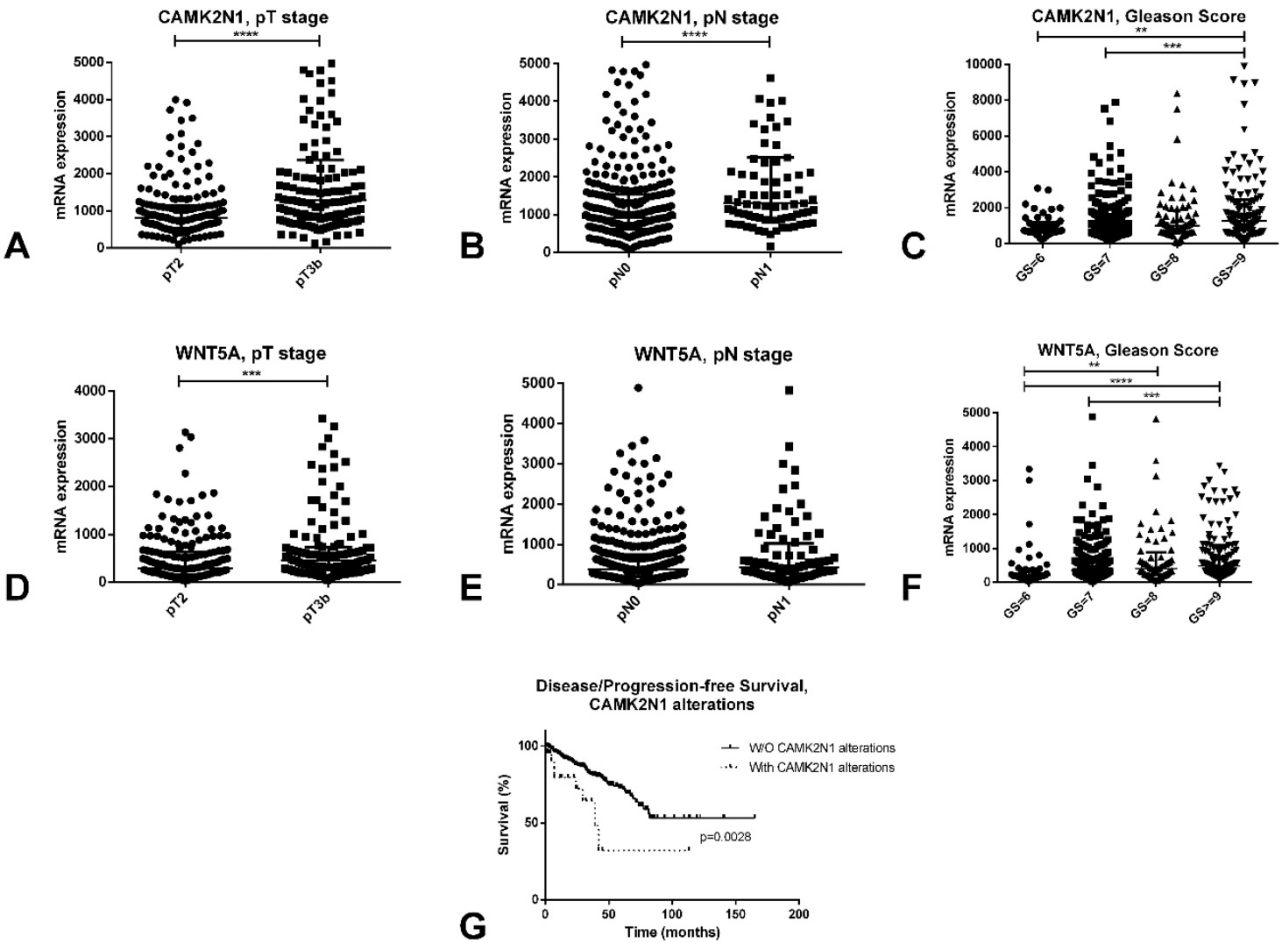
*In silico* analysis of the TCGA database concerning CAMK2N1 and WNT5A. A-C: Differential mRNA expression of CAMK2N1 according to pT stage (A), pN stage (B) and Gleason Score (C). D-F: Differential mRNA expression of WNT5A according to pT stage (D), pN stage (E) and Gleason Score (F). G - Disease/progression-free survival of prostate cancer patients according to CAMK2N1 alterations.

A complete description of the PCa TCGA cohort can be found at 13. Briefly, it includes 498 patients, with a median age at diagnosis of PCa of 61 years. In this series, like in ours, both CAMK2N1 (p<0.0001) and WNT5A (p<0.001) are significantly upregulated in pT3b cases, when compared to pT2 tumors (Figure 5A and 5D). Also, again reproducing results in our cohort, higher Gleason score cases showed significantly higher expression of these players when compared to lower Gleason score tumors (Figure 5C and 5F).

Additionally, CAMK2N1 was found to be significantly upregulated in pN1 tumors when compared to pN0 (p<0.0001), although this was not observed for WNT5A (Figure 5B and 5E). Patients with alterations in CAMK2N1 also displayed significantly worse disease/progression-free survival when compared to those without deregulation of this gene (p=0.0028, Figure 5G).

## Discussion

Increasing the accuracy of predicting the potential clinical aggressiveness of PCa is a major challenge as it constitutes the mainstay to improve patient management and avoid overtreatment. Currently available tools for risk stratification of PCa patients, which include diagnostic serum PSA levels, clinical stage (cTNM) and Gleason score in biopsy 5, 6, are helpful but limited in their ability to accurately predict outcome or tumor aggressiveness 5, 7. Because EMT plays an important role in tumor progression, fostering invasion and metastization 8, we hypothesized that quantitative evaluation of transcript levels of EMT-related genes might allow for improved stratification of PCa patients according to the likelihood of progression to lethal forms of disease.

Thus, we firstly assessed the expression levels of 93 EMT-related genes using a RealTime ready Custom Panel 384 assay, using a limited, though representative, number of PCa tissue samples, compared to normal prostate tissue. Selected tumor samples represented two distinct phases of PCa progression, *i.e.*, small volume, organ-confined disease (pT2a+2b), usually associated with good prognosis, and locally advanced disease (pT3b) which entails high risk of metastization. Although this approach might underestimate alterations in gene expression, it allows for selection of the most significantly altered genes. From array analysis, five candidate genes emerged (*CAMK2N1, CD44, KRT14, TGFβ3* and* WNT5A*) and validation in a larger sample set was mandatory to sanction these preliminary findings. Interestingly, significant differences in gene expression between NPT and PCa were confirmed for *CD44, KRT14* and* WNT5A,* but not for* CAMK2N1* and* TGFβ3.* The results observed for *CAMK2N1* might be explained by the low expression demonstrated by pT2a+b tumors, which is very similar to NPT, negatively impacting in the comparison PCa *vs.* NPT. On the other hand, the opposing results concerning CD44 expression in the array and in the validation series, might derive from the small number of cases analysed in the array and further emphasize the need for validation of array results.

Remarkably, statistically significant associations with pathological stage and Gleason score were disclosed for *CAMK2N1*,* CD44* and *WNT5A*, with more advanced stage and higher GS tumors depicting higher transcript levels. Furthermore, similar results for CAMK2N1 and WNT5A expression were disclosed at protein level, evaluated through immunohistochemistry. Globally, these results indicate that increased expression of those EMT-related genes occurs along tumor progression, which is further underscored by the finding of increased CAMK2N1 and WNT5A immunoexpression in metastatic PCa. Since EMT endows tumor cells with the ability to invade, migrate and metastasize, these findings 8 seem to confirm the hypothesis which set the basis of our study. Interestingly, higher *CAMK2N1* and *WNT5A* transcript levels associated with worse DFS and DSS, although only in univariable analysis. The relatively limited sample size might have impaired the confirmation of an independent prognostic value for both *CAMK2N1* and *WNT5A* transcript levels, but the association with pathological stage and GS, two well established PCa prognostic parameters, is likely also to contribute to this result. Intriguingly, no prognostic value was disclosed for CAMK2N1 or WNT5A immunoexpression, but it should be recalled that immunoexpression analysis is a more subjective and error-prone evaluation than quantitative RT-PCR, which may not be able to capture subtle alterations in gene expression. Moreover, we performed an *in silico* analysis of an independent cohort (as already performed by us 14, 15), which validated our results. Indeed, upregulation of the two most remarkable players associated with pT3b tumors and higher Gleason scores. Additionally, overexpression of CAMK2N1 also associated with pN1 disease, putting in evidence its effect on PCa metastization, and alterations in this gene predicted poorer disease/progression-free survival.

*CAMK2N1*,* CD44* and *WNT5A* have been previously associated with EMT in PCa and other malignancies. CD44, a transmembrane glycoprotein involved in cell adhesion, migration, differentiation, signal transduction and apoptosis 16, 17, has been proposed as a cancer stem cell (CSC) marker in several tumors 18-20, including PCa 21, in which also promotes cell migrations and invasion *in vitro* as well as metastatic dissemination *in vivo* and chemoresistance 21-23. Recently, a CD44+ stem like cell was considered an initiator of EMT contributing to PCa metastasis, suggesting CD44 as poor prognosis marker in PCa 24. Our results are in line with those findings since higher expression levels were found in more advanced and less differentiated tumors.

As for* CAMK2N1*, it encodes for a protein that interacts and inhibits CAMK2 signaling, which is implicated in cell cycle progression through activation of MEK/ERK and Notch-1 pathways 25, 26, both of which have been shown to induce EMT 27. In PCa cell lines, *CAMK2* overexpression induced a decrease in apoptosis, whereas its inhibition reduced proliferation and invasion capacity 28. Thus, considering the actions mediated by CAMK2, a tumor suppressor role would be expected for CAMK2N1. Indeed, there is evidence that CAMK2N1 deactivates MEK/ERK pathway reducing tumor growth 29 and a tumor suppressive role in PCa has been proposed 30, 31. Nevertheless, our findings suggest an oncogenic role for *CAMK2N1* because higher expression was detected in PCa, with significantly increased expression in more advanced tumors and metastases, and association with worse outcome. Survival analysis also demonstrated an association of an increased expression with a poor prognosis. Interestingly, increased *CAMK2N1* expression was found in PCa cases that recurred 32. A possible explanation for these finding is that tumor cells actively engaged in EMT are usually less proliferative 33. Thus, the inhibitory activity of CAMK2N1 on CAMK2 in PCa would provide conditions for cancer cells to endure EMT through slowing cellular proliferative activity. However, the mechanism through which *CAMK2N1* promotes tumor aggressiveness remains unclear and require functional studies.

*WNT5A*, a member of Wnt family, has been implicated in tumor progression and osteomimicky (a process in which prostate cancer cells acquire an osteoblast-like phenotype) 34. Thus, it is expected to play an important role in PCa metastization considering the well-known propensity to metastasize to bone. Wnts signaling pathways are generally classified into canonical/β-catenin dependent pathway and non-canonical/β-catenin independent pathway. WNT5A is one of the most studied non-canonical Wnts, although it has been suggested that, in specific contexts, it may also interact with the canonical pathway 35, 36. Intriguingly, although *WNT5A* has been generally found to be upregulated in PCa, its role is still unclear, as some studies report association with good prognosis 37, 38 whereas other report associations with tumor aggressiveness and poor prognosis 39-41. Indeed, more recent works have shown an implication of *WNT5A* in maintaining PCa cells dormancy in bone 42 and also that lower expression of this player and its receptors FZD5 and RYK associate with improved DSS 43; however, it has also been shown that *WNT5A* induces castration resistant phenotype in the bone niche by interacting with macrophages 44 and that the protein SHISA2 interacts with *WNT5A* in mediating PCa aggressiveness 45. Our findings support the latter hypothesis since we demonstrated *WNT5A* upregulation in PCa, both at transcript and protein levels, associating with higher pathological stage and GS, metastization and worse prognosis.

The relatively small sample size and, to a lesser extent, the follow-up time constitute the main limitations of this study. As previously mentioned, this has most probably impacted negatively in multivariable analysis, precluding the confirmation of CAMK2N1 and WNT5A transcript levels as independent prognostic markers (despite a borderline result for WNT5A and DFS). Moreover, the lack of prognostic significance for CAMK2N1 and WNT5A immunoexpression is also limitative as immunohistochemistry is widely available in Pathology labs, facilitating translation of these findings into routine practice. Nevertheless, our study brought novelty (including methodological novelty) into the study of EMT-related genes in PCa, since it stemmed from a wide, array-based screen of several genes (selected after careful and thorough literature review) in a limited amount of contrasting samples (both normal parenchyma of non-neoplastic prostate glands and PCa samples with distinct aggressiveness), followed by validation in a much larger patient-derived cohort, treated by the same multidisciplinary team of the same institution and, importantly, subjected to the same rigorous pathological assessment. Moreover, besides validation of findings at the transcript level (including survival analyses), the study conclusions were further validated at the protein levels by immunohistochemistry, incrementing their value. Also, inclusion of metastatic samples provided a proof of concept for the study, allowing inferring on the effect of these players on the EMT and metastization phenomenon. Finally, and importantly, main conclusions were further validated in an independent, large and rigorous cohort, the TCGA database, which further strengthens the results.

In conclusion, we demonstrated that two EMT-related genes, *CAMK2N1* and *WNT5A*, are potential biomarkers for assisting in assessment of PCa clinical aggressiveness. Indeed, increased expression was observed in more advanced and less differentiated tumors, as well as in metastases, associating with worse prognosis. These results require further validation in a more extended series of PCa cases, eventually including prostate biopsies to ascertain the ability to convey prognostic information in pre-treatment setting. Nevertheless, validations of the results in an independent cohort (TCGA) further strengthen our conclusions. However, we acknowledge that functional studies might help to further clarify the role of *CAMK2N1* and *WNT5A* in PCa progression.

## Supplementary Material

Supplementary figures and tables.

## Figures and Tables

**Table 1:** Clinical and pathological features of patients included in this study.

	Cohort 1		Cohort 2		Cohort 3		
	NPT	PCa	NPT	PCa	NPT	PCa	Met
Patients, *n*	5	10	16	94	45	94	57
Median Age, *yrs* (range)	61 (54-66)	61 (53-74)	63 (45-80)	63 (50-75)	66 (45-82)	63 (50-75)	
PSA (ng/mL), *median* (range)	*n.a.*	7.75 (4.5-19.9)	*n.a.*	9.75 (2.0-25.4)	*n.a.*	9.75 (2.0-25.4)	*n.a*
**Pathological Stage, *n* (%)**							
pT2a or pT2b	*n.a.*	5 (50.0)	*n.a.*	48 (51.1)	*n.a.*	48 (51.1)	*n.a*
pT3b	*n.a.*	5 (50.0)	*n.a.*	46 (48.9)	*n.a.*	46 (48.9)	*n.a*
**Gleason Score, *n* (%)**							
≤6	*n.a.*	1 (10.0)	*n.a.*	27 (28.7)	*n.a.*	27 (28.7)	*n.a*
3+4=7	*n.a.*	4 (40.0)	*n.a.*	26 (27.7)	*n.a.*	26 (27.7)	*n.a*
4+3=7	*n.a.*	0 (0.0)	*n.a.*	27 (28.7)	*n.a.*	27 (28.7)	*n.a*
8	*n.a.*	0 (0.0)	*n.a.*	5 (5.3)	*n.a.*	5 (5.3)	*n.a*
9-10	*n.a.*	5 (50.0)	*n.a.*	9 (9.6)	*n.a.*	9 (9.6)	*n.a*

NPT, normal prostatic tissue; PCa, prostate cancer; Met, metastatsis; n.a., not available/applicable.

**Table 2:** Values of 2^-ΔΔCt^ between pT3b-PCa and NPT, and pT2a+b-PCa and pT3b-PCa for selected genes.

	2-ΔΔCt	
	pT3b-PCa Vs. NPT	pT3b-PCa Vs. pT2a+b-PCa
CD44	0.16	0.31
KRT14	0.19	0.28
TGFB3	0.21	0.26
WNT5A	2.68	9.16
CAMK2N1	4.76	4.04

NPT, normal prostatic tissue; pT3b-PCa, prostate cancer pathological stage pT3b; pT2a+b-PCa, prostate cancer pathological stage pT2a and pT3b.

## Abbreviations

CAMK2N1: Calcium/Calmodulin Dependent Protein Kinase II Inhibitor 1
CD44: Cluster of differentiation 44
DFS: Disease-free survival
DSS: Disease-specific survival
EMT: Epithelial-to-mesenchymal transition
FFPE: Formalin fixed paraffin embedded
GS: Gleason score
KRT14: Keratin 14
IHC: Immunohistochemistry
MET: Metastatic prostate cancer tissue
NPT: Normal prostate tissue
PCa: Prostate cancer
PSA: Prostate specific antigen
QRT-PCR: Quantitative RT-PCR
SMA: α-smooth muscle actin
TGFβ3: Transforming growth factor, beta 3
TNM: Tumor Node Metastasis staging system
WNT5A: Wingless-type MMTV integration site family, member 5A
