# Supplementary material for: Expression of EMT-Related Genes CAMK2N1 and WNT5A is increased in Locally Invasive and Metastatic Prostate Cancer
Source: J Cancer. 2019 Oct 15;10(24):5915–25. doi: 10.7150/jca.34564 (PMC6856586; doi:10.7150/jca.34564)
Supplement: Supplementary file 1 — Supplementary figures and tables. [file jcav10p5915s1.pdf]

Table S1- Characterization of the 93 potential EMT-related genes selected for this study.

Gene Symbol	Location	Related Functions/Process*
AHNAK	11q12.2	Cell differentiation
AKT1	14q32.32	Cell differentiation and cell proliferation
BMP1	8p21.3	Cell differentiation, extracellular matrix organization and TGFβ pathway
BMP7	20q13	Cell differentiation, cell proliferation and TGFβ pathway
CALD1	7q33	Cell migration
CAMK2N1	1p36.12	Calcium-dependent protein kinase inhibition
CAV2	7q31.1	Cell proliferation and cell migration
CD44	11p13	Cell adhesion and a stem cell marker
CDH1	16q22.1	Cell adhesion
CDH2	18q11.2	Cell adhesion
CDH3	16q22.1	Cell adhesion
CDH11	16q21	Cell adhesion
COL1A2	7q22.1	Extracellular matrix organization
COL3A1	2q31	Extracellular matrix organization and TGFβ pathway
COL5A2	2q14-q32	Extracellular matrix organization
CTNNB1	3p21	Cell morphogenesis, cell proliferation, cell adhesion, cell-extracellular matrix adhesion and WNT pathway
DAB2IP	9q33.1-q33.3	Cell proliferation, cell migrations and Wnt pathway
DSC2	18q12.1	Cell adhesion
DSP	6p24	Cell adhesion, cytoskeleton organization
EGFR	7p12	Cell proliferation, cell migration and cell adhesion
ERBB3	12q13	Cell differentiation, cell proliferation and cell adhesion
F11R	1q21.2-q21.3	Cell differentiation, cell adhesion and TGF pathway
FGFBP1	4p15.32	Cell differentiation, cell proliferation and cell migration
FN1	2q34	Cell migration, cell adhesion and cell morphogenesis
FOXC2	16q24.1	Cell differentiation, cell proliferation, cell adhesion and Notch pathway
FZD7	2q33	Cell differentiation, stem cell division, stem cell maintenance and WNT pathway
GNG11	7q21	Modulation/transduction of transmembrane signaling
GSC	14q32.1	Cell differentiation and Wnt pathway
GSK3B	3q13.3	Cell proliferation, cell migration and WNT pathway
IGFBP4	17q12-q21.1	Cell proliferation
IL1RN	2q14.2	Cell proliferation and cell adhesion
ILK	11p15.4	Cell proliferation, cell adhesion, cell-extracellular matrix, cell migration and Wnt pathway
ITGA5	12q11-q13	Cell adhesion
ITGAV	2q31-q32	Cell proliferation, cell adhesion, cell-extracellular matrix adhesion and cell migration
Gene Symbol	Location	Related Functions/Process*

ITGB1	10p11.2	Cell adhesion, cell-extracellular matrix adhesion and cell migration
JAG1	20p12.1-p11.23	Cell differentiation, cell morphogenesis and Notch pathway
KRT7	12q13.13	Cytoskeleton organization
KRT14	17q12-q21	Cell differentiation, cell adhesion and cytoskeleton organization
KRT19	17q21.2	Cell differentiation and cytoskeleton organization
MAP1B	5q13	Microtubule organization
MITF	3p14.2-p14.1	Cell differentiation
MMP2	16q13-q21	Extracellular matrix degradation and organization
MMP3	11q22.3	Extracellular matrix degradation and organization
MMP9	20q11.2-q13.1	Extracellular matrix degradation and organization, cell-extracellular matrix adhesion and cell proliferation
MSN	Xq11.1	Cell migration
MST1R	3p21.3	Cell differentiation, cell proliferation and cell migration
NKX3-1	8p21.2	Cell proliferation
NODAL	10q22.1	Cell differentiation, cell proliferation, cell migration and TGF pathway
NOTCH1	9q34.3	Cell differentiation, cell morphogenesis and Notch pathway
NUDT13	10q22.1	
OCLN	5q13.1	Cell proliferation and cell adhesion
PDGFRB	5q33.1	Cell proliferation and cell migration
PLEK2	14q23.3	Cytoskeleton organization
POU5F1	6p21.31	Stem cell maintenance and Wnt pathway
PROM1	4p15.32	Cell differentiation, Stem cell maintenance
PTK2	8q24.3	Cell proliferation, extracellular matrix organization, cell adhesion and cell migration
PTP4A1	6q12	Cell differentiation, cell proliferation and cell migration
RAC1	7p22	Cell morphogenesis, cell adhesion, cell-extracellular matrix adhesion, cytoskeleton organization and Wnt pathway
RARB	3p24.2	Cell differentiation and cell proliferation
RASSF1	3p21.3	Cell proliferation and RAS pathway
RGS2	1q31	Cell differentiation and cell proliferation
SERPINE1	7q21.3-q22	Cell adhesion and cell migration
SFRP1	8p11.21	Wnt pathway
SIP1	14q13	Transcription factor
SMAD2	18q21.1	Cell differentiation, cell proliferation, cell morphogenesis and TGFβ pathway
SMAD4	18q21.1	Cell differentiation, cell morphogenesis and TGFβ pathway
SNAI1	20q13.2	Cell differentiation, cell morphogenesis, cell migration and Notch pathway
**Gene Symbol**	**Location**	**Related Functions/Process***
SNAI2	8q11	Cell differentiation, stem cell proliferation, Notch pathway and Wnt pathway
SNAI3	16q24.3	Transcription factor
SOX10	22q13.1	Cell differentiation, cell proliferation, cell morphogenesis and Wnt pathway

SPARC	5q31.3-q32	Cell morphogenesis, Extracellular matrix organization and cell migration
SPP1	4q22.1	Cell adhesion and cell-extracellular matrix adhesion
STAT3	17q21.31	Cell proliferation, cell migration and Notch pathway
STEAP1	7q21	Cell adhesion
TCF3	19p13.3	Cell differentiation, cell proliferation and transcription factor
TCF4	18q21.1	Cell differentiation and transcription factor
TFPI2	7q22	Extracellular matrix component
TGFB1	19q13.1	Cell proliferation, cell migration, cell adhesion and TGFβ pathway
TGFB2	1q41	Cell differentiation, cell proliferation, cell morphogenesis, cell migration, cell adhesion and TGFβ pathway
TGFB3	14q24	Cell differentiation, cell proliferation, cell adhesion and TGFβ pathway
TIMP1	Xp11.3-p11.23	Cell proliferation, cell migration, cell adhesion and TGFβ pathway (inhibitor of MMP's)
TMEFF1	9q31	Cell differentiation
TMEM132A	11q12.2	Cell differentiation
TSPAN13	7p21.1	Cell migration
TWIST1	7p21.2	Cell differentiation and transcription factor
VCAN	5q14.3	Cell proliferation, extracellular matrix component, cell adhesion and migration
VIM	10p13	Cell migration and cytoskeleton organization
VPS13A	9q21	Proteins transport
WNT11	11q13.5	Cell differentiation, cell proliferation, cell migration and WNT pathway
WNT5A	3p21-p14	Cell differentiation and WNT pathway
WNT5B	12p13.3	Cell differentiation, cell migration and WNT pathway
ZEB1	10p11.2	Cell differentiation, cell proliferation and transcription factor
ZEB2	2q22.3	Transcription factor

* Based on information available in *http://www.ncbi.nlm.nih.gov/*

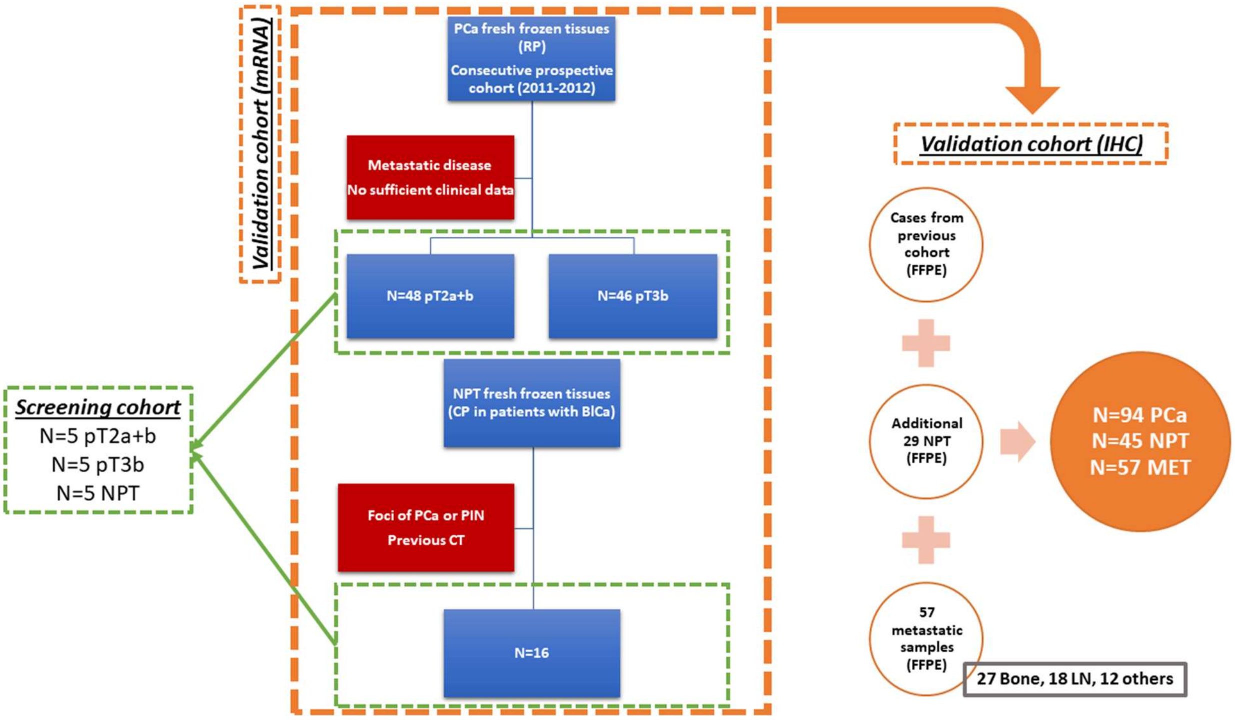

Validation cohort (mRNA)
PCa fresh frozen tissues (RP)
Consecutive prospective cohort (2011-2012)
Metastatic disease
No sufficient clinical data
N=48 pT2a+b
N=46 pT3b
NPT fresh frozen tissues (CP in patients with BlCa)
Foci of PCa or PIN
Previous CT
N=16
Screening cohort
N=5 pT2a+b
N=5 pT3b
N=5 NPT
Validation cohort (IHC)
Cases from previous cohort (FFPE)
Additional 29 NPT (FFPE)
57 metastatic samples (FFPE)
27 Bone, 18 LN, 12 others
N=94 PCa
N=45 NPT
N=57 MET
